# Perspective from anesthesiologists on the therapy of critically ill patients with COVID-19

**DOI:** 10.1007/s44254-023-00009-3

**Published:** 2023-03-10

**Authors:** Hui Li, Ruping Dai

**Affiliations:** Department of Anesthesiology, the Second XiangYa Hospital, Central South University, ChangSha, China

**Keywords:** COVID-19, Critically ill, Therapy, Anesthesiologists, Perspective

## Abstract

With the surge of critically ill COVID-19 patients in China, numerous anesthesiologists from anesthesia intensive care units (AICU) or reallocated to other ICUs were devoted to the treatment of COVID-19. Besides the standard protocols to treat COVID-19 cases, anesthesiologists also have their own experience to treat COVID-19 cases based on professional expertise and practice. Here, we propose some viewpoints to treat critically ill COIVD-19 patients from the perspective of anesthesiologists.

Since the optimization of COVID-19 virus control strategy in early December, COVID-19 cases have increased dramatically, forcing many hospitals to reallocate their resources to address the surge of patients requiring intensive care. In this context, the hospital activities perceived as non-urgent, such as selective surgeries, were decreased or indeed suspended. Faced with the surge of critically ill COVID-19 patients, all the intensive care units (ICU) including Anesthesia ICU (AICU) hosted the critical COVID-19 cases. A number of anesthesiologists in China were reallocated to AICU or other ICUs and actively treated the severe cases. Standard protocols to treat COVID-19 cases have been implemented in various ICUs including prone position ventilation, anti-COVID small molecular therapy, low dose corticosteroids treatment and so on [1]. However, there are still some subtle distinctions in the treatment of critically ill cases in different ICUs, based on the professional background and skills of physicians from different ICUs. In this regard, anesthesiologists have great expertise in analgesia and sedation, hemodynamic monitoring and airway managements, and have unique viewpoints into the treatment of critically ill COVID-19 patients. Looking back on the past spike of omicron infections, here we summarize some of our experiences of treating COVID-19 cases from an anesthesiologist’s perspective.

## The application of nasotracheal intubation in AICU

Endotracheal intubation is the prerequisite for mechanical ventilation. Orotracheal intubation is also widely used in most ICUs. As anesthesiologists, we recommend nasotracheal intubation for the patients requiring long-term intubation. Compared to orotracheal intubation, nasotracheal intubation has several advantages for the treatment of critically ill patients with COVID-19. First, it improves the tolerance of long-term intubation for these patients and greatly reduces the doses of sedatives and analgesic drugs required, which may ultimately lead to circulation instability and tissue hypoperfusion. Second, it reduces the difficulty of care for COVID-19 patients: Nasotracheal intubation is easy to fix and oral care can be performed. There is no need to place an oral plug, which avoids tongue swelling, ulcers and crush necrosis caused by long-term insertion. Third, the patients are more tolerant during the procedure of weaning from the mechanical ventilation. For critically ill patients with COVID-19, weaning from the mechanical ventilator is a long procedure and requires the patient’s cooperation. The high tolerance of nasotracheal intubation allows patients to be cooperative and this is very helpful for weaning.

## Dorsal lung region: the critical zones of lung ultrasound (LUS) examination

**Fig.1:**
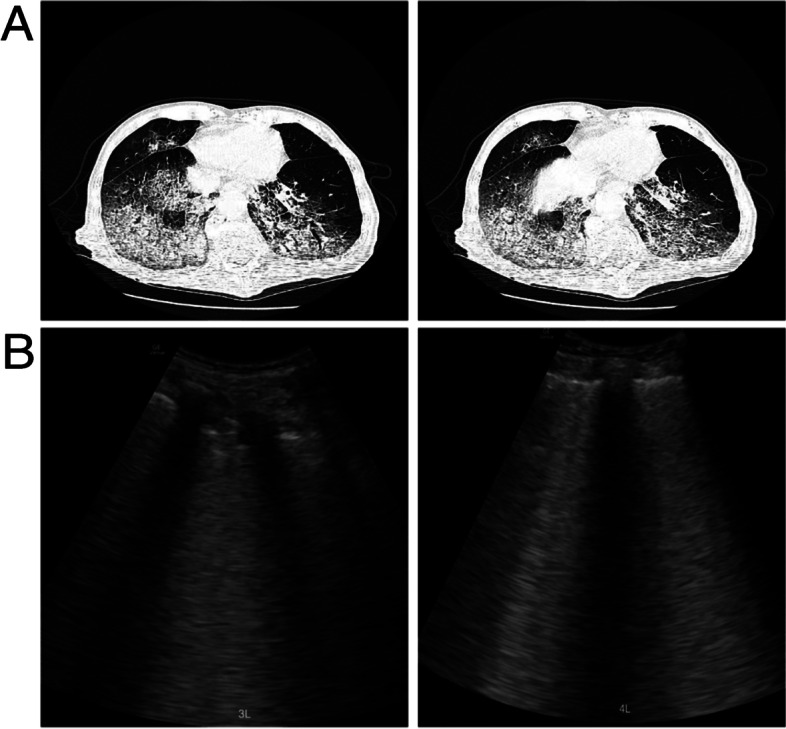
CT and LUS examination of a 78-year-old male COVID patient. **A** CT images showing the changes in the subpleural-peripheral in the dorsal zones of both lungs. **B** LUS shows multiple coalescent B-lines visible during the prone ventilation

As an advantageous bedside-available and point­of­care imaging tool to support clinical decision-making, LUS allows for a quick evaluation of various lung alterations, including pulmonary edema, lung inflammation, pleural effusion and pneumothorax [2]. The 8-zone protocol is usually performed in supine patients. However, the pathological changes in critically ill COVID patients mostly present on the dorsal region, which 8-zone LUS can easily neglect. Instead, a 12-zone protocol allows a comprehensive whole-organ assessment to quantify the level of involvement. For patients undergoing prone position ventilation, assessing posterior regions may provide more information consistent with the CT imaging characteristics, such as disease severity, therapeutic effect and prognostic outcomes (Fig. 1).

## Minimally invasive hemodynamic monitoring: a powerful strategy to assess cardiac function and volume management

With the use of lung-cardiac-inferior vena cava (LCI) integrated ultrasound, the alterations of lung edema, inflammation, the severity of volume overload and cardiac function, particularly right ventricle abnormalities with an incidence of 39% observed in COVID-19 [3], can be rapidly and accurately identified. But its learning curve is relatively long, posing a great challenge for anesthesiologists who were previously unfamiliar with ultrasound imaging technology. In contrast, anesthesiologists are experts in monitoring hemodynamics, with the use of minimally invasive instruments such as proAQT and Vigileo. Hemodynamic parameters including stroke volume variability (SVV), cardiac index (CI), peripheral vascular resistance index (SVRI) provide valuable information about cardiac function and fluid responsiveness. When combined with LCI integrated ultrasound, continuous hemodynamic monitoring further confirms the ultrasound assessments and provides additional information, guiding the anesthesiologists to better management of cardiopulmonary functions.

## Delivery of nutritional support through a nasointestinal tube

For critically ill COVID patients, the application of early enteral nutrition can improve their nutritional status and enhance immune functions. However, 50%–60% of critically ill patients are prone to gastroparesis, causing gastroesophageal reflux and increasing the risk of aspiration pneumonia, especially when they are in the prone position [4]. In addition, administration of analgosedation for patients undergoing mechanical ventilation can also slow gastric emptying. A nasointestinal tube can directly deliver nutrients to the duodenum with low gastric residual volume, conferring protection from aspiration caused by reflux and enhancing feeding tolerance.

For diabetic patients, attention should be paid to the fluctuation of blood glucose concentrations during enteral nutrition, which is a frequent complication of enteral nutrition that increases the risk of complications and mortality. If insulin therapy is required, it is preferred to continuously infuse insulin with an infusion pump as it allows frequent dose adjustments accurately to control plasma glucose concentrations. In an attempt to let critical care nurses understand the protocol of adjusting glucose concentrations, we suggest that the protocol is written in large font on the bedside and confirm that the nurses absorbed the knowledge. Generally, it is required to start insulin pump infusion or increase the infusion speed during the period of enteral nutrition to prevent hyperglycemia. When the enteral nutrition ceases, the amount of pump injection must be reduced or stopped to prevent severe hypoglycemia caused by abrupt discontinuation of nutrition support. The latter procedure is very important since it is easy to forget to adjust the infusion speed when enteral feeding is over, which may result in hypoglycemia. It is also important to ask the nurse to closely monitor the glucose concentrations and to adjust the infusion speed in a timely manner, especially in the circumstances of nurse shortage and job overload.

## Individualized mechanical ventilation strategy by real-time clinical evaluation

Lung-protective ventilation is the featured treatment of critically ill patients with COVID-19. Despite of the beneficial effect of oxygenation and mobilization of bronchial secretions in ARDS patients, prone ventilation easily renders the resistance to ventilation, particularly in patients with multiple comorbid conditions [5, 6]. Hence, individualized mechanical ventilation strategies are proposed for COVID-19 patients. The ideal ventilation mode can reduce the workload of lung and heart, thereby beneficial for lung repair and reduction of myocardial oxygen consumption [7]. Anesthesiologists are adept at bedside and real-time evaluation for patients with the use of point-of-care ultrasound. They can adjust the ventilator parameter timely based on the patients’ situations, thus providing optimal individualized mechanical ventilation strategies. This would help the critically ill patients accelerate the weaning process and rehabilitation.

Anesthesiologists have the ability and should play active roles in treating severely ill patients with COVID-19 when there are insufficient emergency and ICU physicians available. Through active participation in caring for critically ill patients with COVID-19, the capacity of anesthesiologists to cope with critically ill patients improves greatly with experience. Anesthesiologists may also be able to provide constructive comments on the treatment of critically ill patients and properly fulfill this role for contribution to their prognosis after being infected with COVID-19.

## Data Availability

No.

## Abbreviations

AICU: Anesthesia intensive care units
ICU: Intensive care units
LUS: Lung ultrasound
LCI: Lung-cardiac-inferior vena cava
SVV: Stroke volume variability
CI: Cardiac index
SVRI: Peripheral vascular resistance index
